# Supplementary material for: Big Data in metagenomics: Apache Spark vs MPI
Source: PLoS One. 2020 Oct 6;15(10):e0239741. doi: 10.1371/journal.pone.0239741 (PMC7537910; doi:10.1371/journal.pone.0239741)
Supplement: Attachment — Submitted filename: ResponseToReviewers.pdf [file pone.0239741.s001.pdf]

Big Data in Metagenomics: Apache Spark vs MPI

José M. Abuín, Nuno Lopes, Luís Ferreira, Tomás F. Pena, Bertil Schmidt

Response to reviewers

We would like to thank the reviewers for their insightful comments and suggestions about the paper. All the changes in the revised manuscript are highlighted in red. Detailed responses to reviewers are given below (`black font`: original reviewer comments, **`blue font`**: responses).

```
##############################################################
##############################################################

Reviewer #1:
I think that the authors deals with an important and interesting topic but
the thing that they do is not really novel and many key paper in literature
are not even mentioned (e.g. [1]) which does a very similar thing but with
also the addition of OpenMP to MPI.

[1] Reyes-Ortiz, J. L. and Oneto, L. and Anguita, D., INNS International
Conference on Big Data (INNS BIG DATA),
Big Data Analytics in the Cloud: Spark on Hadoop vs MPI/OpenMP on Beowulf,
2015.
```

We agree with the reviewer that there are more works that deal with similar problems and we have added the given reference. However, we were not able to find any similar comparison related to metagenomics or Animal Food Sequencing (AFS), so we could not add other references.

```
Reviewer #2:
The authors use MPI to implement an existing algorithm and clearly
demonstrate that the MPI implementation is more efficient than the
original Spark version. The result is clearly applicable to the current
food sample processing pipeline and also demonstrates the room for
improvements for Spark applications in general.
```

Indeed, as the reviewer says, there is room for improvements in general Spark applications related with genomics and other scientific problems. To show this is, in fact, one of the main objectives of this paper.

```
----------------------------------------
```
